# Supplementary figures and images for: Pollen identification through convolutional neural networks: First application on a full fossil pollen sequence
Source: PLoS One. 2024 Apr 30;19(4):e0302424. doi: 10.1371/journal.pone.0302424 (PMC11060525; doi:10.1371/journal.pone.0302424)

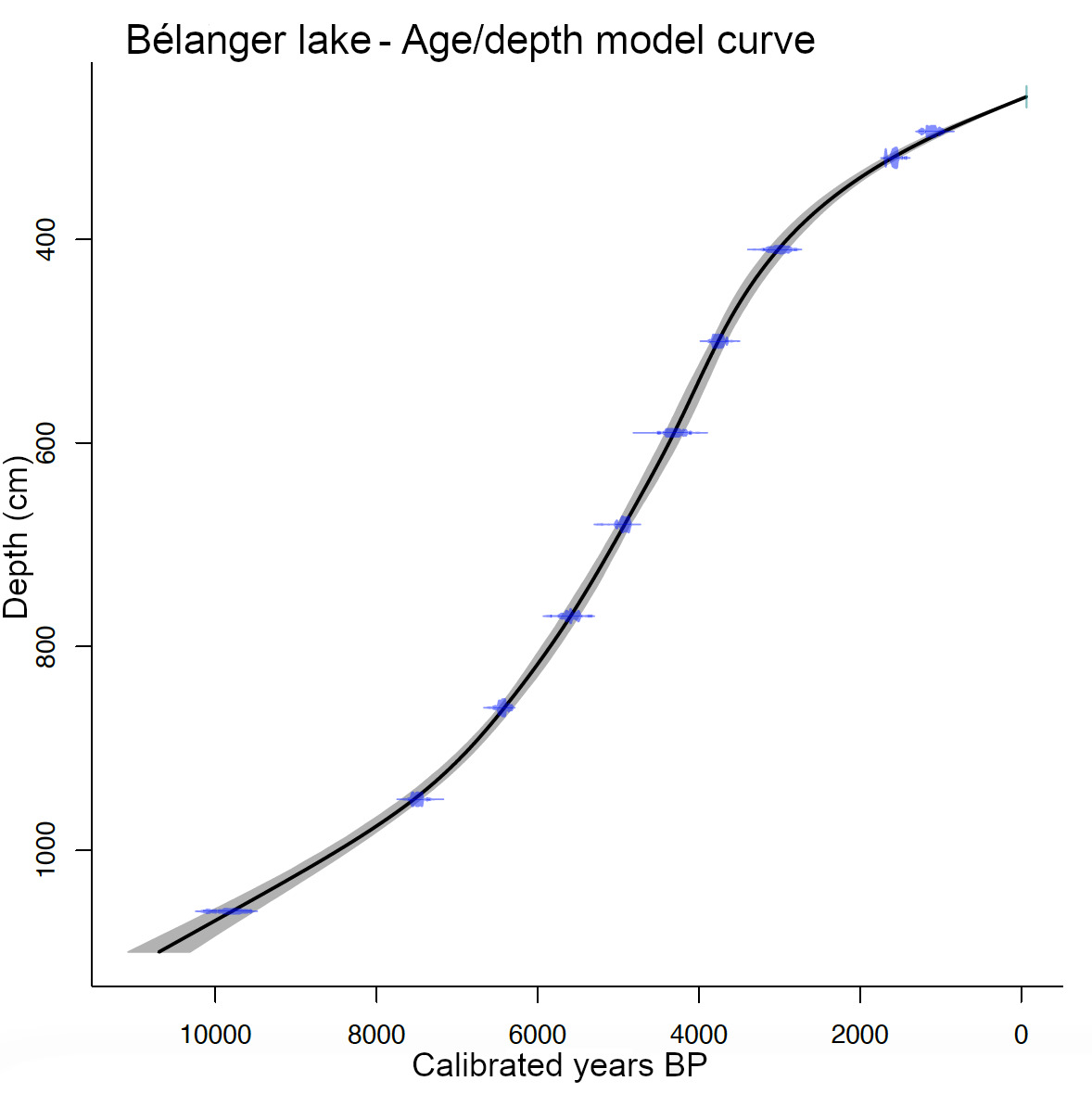

Supplement: S1 Fig — 10 samples of bulk gittya bulk were dated. The blue lines represent the samples’ age and their confidence error. The age/depth model was achieved using a smooth-spline function. (JPEG) [file pone.0302424.s001.jpeg]
