# Supplementary material for: Pollen identification through convolutional neural networks: First application on a full fossil pollen sequence
Source: PLoS One. 2024 Apr 30;19(4):e0302424. doi: 10.1371/journal.pone.0302424 (PMC11060525; doi:10.1371/journal.pone.0302424)
Supplement: Attachment — Submitted filename: response_to_reviewers.pdf [file pone.0302424.s003.pdf]

Pollen identification through convolutional neural networks: First application on a full fossil pollen sequence

Review comments to the authors

Comments from the Academic Editor

Please ensure that your manuscript meets PLOS ONE's style requirements, including those for file naming.

- *The revised manuscript now meets the style requirements. In particular, this is in regard to file naming, SI naming, the bibliography and the heading font size.*

We note that the grant information you provided in the 'Funding Information' and 'Financial Disclosure' sections do not match.

- *As established in the additional comments section of the Submission system: "The funding acquired through the Ministère des Ressources naturelles et des forêts (Québec) has been attributed in your submission system to the "Ministère des Ressources naturelles et de l'énergie". The latter does not exist anymore, but we entered it as such because the former does not exist within your system as of yet."*

We note that Figure 1 in your submission contain map/satellite images which may be copyrighted.

- *The map in figure 1 has been created by a credited team member (Véronique Poirier) working for the Québec provincial government. She uses data available through the provincial government databases – contour lines and forest zones, as well as waterbodies. The database is called Forêt-Ouverte (open forest), more info can be found here (In English):* *https://mffp.gouv.qc.ca/documents/forest/user_guide_foret_ouverte.pdf*

- *And here, in French, which states that the database is under a CC*-BY 4.0 Licence: https://www.donneesquebec.ca/recherche/dataset/carte-ecoforestiere-avec-perturbations/resource/7ada89ee-0d80-4c6a-b4e3-1090b18e34b8

- *Many papers have been published featuring maps that use the same governmental databases without any copyright issues. Pierre Grondin, who just in case has signed the copyright form allowing for use of this data, is a researcher for the Natural Ressources and Forest Ministry of Québec (MRNF).*

Comments from Reviewer #1:

The abstract lacks open questions and limitations of the AI existing methods.

- *An additional line concerning a limitation of AI methods in fossil palynology has been added to the revised manuscript. We otherwise believe limitations concerning existing AI methods are already listed in the Abstract: "Still, only a small portion of works published on the matter address the classification of fossil pollens"; "[…] there exists a gap between the training data and the target data".*

Figure 2 is the main one is fully unclear and not able to judge it. Please redraw it.

- *Unfortunately, we do not see how to more efficiently convey the information presented in Figure 2. This figure allows the reader to understand the hierarchical aspect of our method. We believe the color coding and legend help the readers to work their way around the figure. The white boxes give relevant information on the CNNs' parameters for those willing to play around the model. The blue boxes give important information to paleoecologists versed in Northeastern American pollens interested in knowing which species were used in forming the initial training classes.*
- *We tested moving the white boxes into a separate table, but they are hardly separable – understanding the content of the white boxes requires seeing the tree-like structure and how the CNNs relate to one another. Since this figure is referred to many times throughout the manuscript, this slowed down the reading process.*
- *We did, however, add a bit more meat onto the figure description in order to further guide the reader.*

Contributions of the paper should be listed at the end of introduction.

- *This has been added to the revised manuscript.*

Lack of statistical evaluation. Please list them as a table

- *A table has been added to the revised manuscript (Table 3)*

Paper presentation has to be approved Such as adding Grad-Cam.

- *We believe that adding Grad-cam visualization would burden an already elaborate and multi-faceted manuscript. Grad-cam is seldom used in other papers pertaining to pollen classification networks.*

List the issues with previous methods that this paper solved in related work.

- *The performance of our method is compared to the performance of other similar methods in the discussion. However, since our method is the first of its kind to be trained*

on pollens found in Quebec organic fossil records, it cannot directly solve issues found in related works as: a) their pollen data come from plant assemblages not endemic to Quebec; and b) no other paper – that we know of – has tackled the challenge of classifying pollens found in organic fossil records. We emphasize the fact that the goal of this manuscript is not so much displaying new methods in pollen classification networks as it is creating a freely available tool for palynologists working on Quebec fossil pollen records. We do, however, list the issues with previous methods in traditional (Non-AI) palynology in the abstract and introduction sections.

Comparison with recent previous methods (2021-2022-2023) in terms of results on same used datasets with proper citation of previous methods. I would suggest validating the proposed solution on more than one public dataset.

- *Comparing our method and its results to other published methods is not possible. This is because our method is only able to classify the pollens and objects most commonly found in Quebec organic sediment records. In other words, no public models can classify our target pollens and we cannot classify the target data of other pollen classification models. No labelled dataset suited for our topic currently exist. This is the reason why we had to resort to validating our results using human palynologist predictions (a time intensive process that was necessary in order to achieve any kind of data validation). We thus cannot validate our method on any other public dataset or compare it any further than we already have on recent published methods.*

Comments from reviewer #2:

For example, it is unclear why choosing to "transformed to grayscale and rescaled to 128x128 pixels" (Line 197-8) would standardize the data and improve training performance. This more likely prevented further improvement using RGB colors and larger resolutions. It would be better to just say "we transformed ... in this work".

- *This has been changed in the revised manuscript.*

The choice to limit each CNN's corresponding dataset size to the number of images present in least populated class" (Line 221), throws away a large amount of valuable data. Training on imbalanced data is an established topic in computer vision and there are alternate approaches. A more natural solution would be to adopt weighted loss which assigns a larger weight to rare class.

- *We recognize that neglecting to train the data on imbalanced data was an oversight – we then believed that diving into weighted loss methods would fall outside of the scope of the paper. This is now addressed in the conclusion of the revised manuscript, and will be identified as something to add onto future work.*

Lines 241-246 mentions "transfer learning" but this is actually a discussion of network architecture. The authors should explain how the network was pretrained and on what data, such that the weights can be transferred to pollen data.

- *We included more information on VGG16 and the ImageNet dataset in the revised manuscript. In the quoted paragraph, a few lines already establish how VGG16 can help our own model extract basic features from our data, with references to other papers in automated palynology. We added a line to further explain this, as well as a reference to Alzubaidi et al. (2021).*

The choice to train multiple CNNs was unusual – including the use of a shallow network to train the Alnus CNN because "(deeper models) tend to overfit rapidly" (Line 268). Why did the authors not train a single CNN backbone shared by all classes using all data (allowing learning richer features for all classes)?

- *The inherent taxonomic nature of pollens allowed us to work in a hierarchical classification system (as seen in figure 2). This permits us to have a pollen drop out of the classification and be classified as at the genus level if it falls under a confidence threshold, as seen in Bourel et al. (2020). Similarly to their work, we trained multiple, smaller, CNNs instead of a single one.*
- *Training a single CNN would have us face the issue of an important data imbalance (further addressed in the now revised conclusion) since certain species-level classes had ten times the amount of training data than some others. On the other hand, this allowed us to capitalize on the large amount of training data available when grouping certain*

species in taxonomic classes. For instance, the top classifier trained on 1,200 images per class.

- *As proposed in the reviewer comment, something that could have been done is using a CNN trained on all classes and using its base layers in transfer learning. This is now addressed in the revised conclusion.*

Overall, the machine counts did not appear to closely track human/manual counts. The authors make two interesting choices in their comparison. First – human counts are made from images and not from a microscope. This should remove the effect of poor-quality images, as both human and machine classifications would be affected. But there is still low correlation between the two counts outside of a handful of taxa, e.g. Pinus (Figure 7). Having the human analysis identify detected grains/cropped images doesn't test whether all pollen are identified by the Classifynder, or if there is any systematic bias in what is automatically imaged/detected. Second – given that this is a proof-of-concept paper, why not manually count all 271 samples so that the manual and automatic counts and zonation could be more accurately compared?

- *We indeed chose to validate our model's results using images captured by the Classyfinder microscope. This is addressed in the original manuscript (line 392). In short, this was the only way to isolate our model's own error and bias. What we propose in this paper is not a fully integrated system that requires the Classifynder microscope. Rather, it is a classification tool fit for any fossil pollen sequence whose images have been digitized using any form of light-microscope, automated or not.*
- *Second – while this would have improved the proof-of-concept aspect of our manuscript, counting the full 271 samples would imply counting and identifying a little under 200,000 images. A large part of those images do not belong to any of our trained classes – a single sample can often contain pollen grains and spores from more than 30 species. Since pollen identification is a tedious process and requires a trained specialist, this fell outside of the time and financial limits of the project.*

Based on the images in Figure 3, the discussion of image quality (Lines 526-9) should be significantly expanded. It may be the quality of the images that are the issue and not your models. See the following papers on what is possible with higher quality images:

Punyasena, S. W., Tcheng, D. K., Wesseln, C., & Mueller, P. G. (2012). Classifying black and white spruce pollen using layered machine learning. New Phytologist, 196(3), 937-944.

Kong, S., Punyasena, S., & Fowlkes, C. (2016). Spatially aware dictionary learning and coding for fossil pollen identification. In Proceedings of the IEEE Conference on Computer Vision and Pattern Recognition Workshops (pp. 1-10).

Romero, I. C., Kong, S., Fowlkes, C. C., Jaramillo, C., Urban, M. A., Oboh-Ikuenobe, F., ... & Punyasena, S. W. (2020). Improving the taxonomy of fossil pollen using convolutional neural networks and superresolution microscopy. Proceedings of the National Academy of Sciences, 117(45), 28496-28505.

- *We are thrilled to see how super high-resolution confocal/fluorescence imaging can be integrated into deep learning methods with such good results. We wonder if the above mentioned methods using Zeiss products would be operable for high-volume imaging? Nonetheless, this further shows how imaging may have had a bigger impact on our model than previously thought. We have expanded the discussion of image quality in the revised manuscript.*

L 55: Mander et al 2014 (citation #4) focuses on the classification of grass pollen, which is notoriously difficult to tell apart, even under electron microscopy. It is not the best example of the reproducibility of visual pollen identification and puts traditional palynological data collection in a more negative light than necessary.

- *This is a valid concern. We revised the manuscript in consequence, and referenced other works that touch on the general coherency between analysts.*

L 56: McLeod et al 2010 does not focus on palynology. It also predates the bulk of machine learning research in palynology. There are better references that would characterize the current state of palynological research.

- *We revised the manuscript in consequence; Holt & Bennet (2014) and Mander & Punyasena (2018) give much more accurate insight on the shortcomings of pollen analysis in their respective review papers. Both identify the possible benefits of automated palynology.*

L 65: A number of papers have discussed slide scanning and can be cited here, including:

Holt, K. A., & Bebbington, M. S. (2014). Separating morphologically similar pollen types using basic shape features from digital images: A preliminary study. Applications in Plant Sciences, 2(8), 1400032.

- *In the revised manuscript, cited works related to introducing a deep-learning/slide-scanner workflow, including Holt and Bebbington (2014), Sevillano, Holt & Aznarte (2020) and Pedersen et al (2017).*

L 70, L 90-95: Punyasena et al 2022 (citation #8) also used a slide scanner and image segmentation/pollen detection instead of manual crops. While these were environmental pollen samples (from pollen traps), they have similar issues to Quaternary pollen samples.

- *L70: Added Punyasena et al 2022 to the other citations.*
- *L90-95: Cited Punyasena et al 2022 as an example of combining image detection and an automated slide scanner in the revised manuscript.*

L 173: Were vouchers collected to confirm and record species identifications?

- *We are regretfully unsure of the meaning of this question. The chosen tree species are handily identifiable in the wild and are not known to present a challenge in the matter.*

L 190: Provide more detail on how "pollen was automatically identified through the use of morphological criteria input by the user". Is this using the Classyfinder software? If so, explain how this is automated and what specific criteria are used.

- *This was using the Classyfinder software. We added the specific criteria in the SI.*

L 192: Are you using a Z-stack (multiple images) of pollen specimen or a single fused image? This is not clear.

- *This was using a single fused image. Line has been reworked to reflect this in the revised manuscript.*

L 208: The GitHub repository mentions that the Python code should work with PyTorch. Might be worth mentioning that here too.

- *Added to the revised manuscript.*

L 241: What is meant by the "bottom" of the model? The initial layers?

- *The initial layers. Line has been revised in the new manuscript.*

L 299: Using different processing methods for reference pollen, fresh pollen, and fossil pollen introduces visual artifacts that makes the classification problem much harder than it should be. Ideally, as many variables as possible should be controlled. The authors should consider comparing like with like in their training and testing and explicitly test whether processing choices affected the results.

- *There is regretfully no way around processing the fresh and fossil pollens differently. This is addressed in section 2.4, as well as what laboratory methods were used to minimize discrepancy. The goal of this project was to create a model capable of classifying fossil pollens extracted from a sediment core. The fossil pollens unavoidably undergo a different processing method than fresh pollens do. This is mostly in order to flush out siliceous material (dense-media separation, sodium polytungstate), de-clump the pollens and burn the remaining organic particles (filtering, KOH and ultrasonic bath). Most of the remaining visual artifacts were filtered out using the Main CNN, which has a very*

accurate NPP/mineral class. Fresh pollens do not need to undergo such a process – instead, some of them require to have their inside cleaned out of cellular content using either enzymes or acetolysis.

- *We do not believe that training on 14 classes (and 18 species) would have been possible using fossil pollens – this would imply a point and click method of gathering data going through a fossil pollen slide. This would not have allowed us to gather as much data. Furthermore, a training set composed of fossil pollens found on a slide would add bias: only the pollens that are in good condition with apparent features would have been chosen, in order to avoid wrongly labeling a pollen.*

- *Using fresh pollens allowed us to train the model on considerably more images than what we could have gotten by individually identifying and labelling fossil pollens. This also allowed us to collect data of heavily damaged pollen (of such that is commonly found in organic (lacustrine) sediments, for instance the often found saccus of an unknown* Pinus *or* Picea *grain floating about). Such pollens would have otherwise proved to be impossible to visually identify and add to the training set (Does this saccus belong to a* P. banksiana*,* P. strobus or P. resinosa?).
- *This rationale has been added to the revised manuscript (Line 118) The lab protocols can be found in sections 2.2 and 2.4 and we invite other researchers to experiment and improve it.*

L 326: As per the comment above, any visual documentation of the differences in this alternate processing method?

- *We believe this falls outside the scope out the paper and would only burden and already elaborate manuscript. We do however plan on submitting this lab protocol, along with visual documentation, in the future – we believe its output is impressive (30 organic sediment samples in a day is higher than our usual 16-20) and would benefit from being be tested in other labs.*

L 553: The word "recrudescence" has negative connotations. Use "re-emergence" or "reappearance" instead.

- *This has been changed in the revised manuscript.*

Recurring: Recommend using a spelling/grammar check to catch errors. Species and genera names should be italicized.

- *We understand that this italicizing (or lack) may be an issue. We made sure to only italicize species and genera names – the names of classes are not. This helps differentiate when we write about the species or their respective model classes. Model classes are*

often comprised of multiple species but are labelled as being one, as is sometimes done in palynology: for instance, the Picea class is made up of Picea glauca and Picea rubens.

- *We added a disclaimer in the description of Table 2.*

Figure 3: Include example images of all 13 classes.

- *The figure has been updated in the revised manuscript, along with the figure legend.*

Figures 8 and 9: It would be helpful to see zonations overlaid or side by side.

- *Figure 9's zonations are already overlaid on figure 8 (dotted-lines). This is stated in the figure description.*
