# Supplementary material for: Pollen identification through convolutional neural networks: First application on a full fossil pollen sequence
Source: PLoS One. 2024 Apr 30;19(4):e0302424. doi: 10.1371/journal.pone.0302424 (PMC11060525; doi:10.1371/journal.pone.0302424)
Supplement: Attachment — Submitted filename: response_to_reviewers.pdf [file pone.0302424.s004.pdf]

Pollen identification through convolutional neural networks: First application on a full fossil pollen sequence

Response to reviewers

NB: The authors' answers are in *italics*.

<u>Comments from Reviewer #1:</u>

"The authors did not put enough effort to improve the paper
The comments haven't addreesed very well, for example
1- The quilty of figure 2 very poor, I can't read what was written
2- it is not clear what is the novely of the paper
3- please go back to main comments and address them."

- *Since this refers to comments made during the first round of review, we have again addressed those previous comments in the manuscript. We detail our answers below:*

The abstract lacks open questions and limitations of the AI existing methods.

- *An additional line concerning a limitation of AI methods in fossil palynology has been added to the abstract. Line [25-26]: "*However, Convolutional Neural Networks require a large amount of data for training and databases of fossilized pollen are rare and often incomplete." *This line highlights both a well-known limitation of AI methods, but also specifies particular limits to automated pollen analysis (the lack of fossil pollen databases). Other limitations concerning existing AI methods are also listed in the Abstract, Line 20: "*Still, only a small portion of works published on the matter address the classification of fossil pollens"; Line 27: "[…] there exists a gap between the training data and the target data*".*

Figure 2 is the main one is fully unclear and not able to judge it. Please redraw it.

- *Figure 2 has been redrawn. Some of it has been moved to a new table (Table 3).*

Contributions of the paper should be listed at the end of introduction.

- *Contributions of the paper are listed at the end of the introduction, Line 182.*

Lack of statistical evaluation. Please list them as a table

- *A table has been added to the revised manuscript (Table 3). It is referenced throughout the manuscript.*

Paper presentation has to be approved Such as adding Grad-Cam.

- *We still believe that adding Grad-cam visualization would burden an already elaborate and multi-faceted manuscript. Grad-cam is seldom used in other papers pertaining to pollen classification networks.*

List the issues with previous methods that this paper solved in related work.

- *The performance of our method is compared to the performance of other similar methods in the discussion: Lines [678-684].*
- *However, since our method is the first of its kind to be trained on pollens found in Quebec organic fossil records, it cannot directly solve issues found in related works as: a) their pollen data come from plant assemblages not endemic to Quebec; and b) no other paper – that we know of – has tackled the challenge of classifying pollens found in organic fossil records. We do, however, list the issues with previous methods in traditional (Non-AI) palynology in the abstract and introduction sections.*
- *We list related work and what our project does differently in the introduction, lines [111-146].*
- *This manuscript is a novelty – As stated in the title, this is the first application on a full fossil pollen sequence. Other methods cannot classify full fossil pollen sequences. Our project is a proof of concept. This is highlighted in the abstract (Lines [26-31, 36-38], in the introduction Lines [97-109, 126-138] ("*Despite all of this work, there are no deep learning studies that trained a CNN that is adapted to the large-scale classification of fossilised pollen."; "*This paper aims to train a CNN adapted to the classification of images of fossilised pollens found in Québec organic sediment cores* ")

Comparison with recent previous methods (2021-2022-2023) in terms of results on same used datasets with proper citation of previous methods. I would suggest validating the proposed solution on more than one public dataset.

- *We did not change our answer to this comment since the last review. This comment is not applicable to our project. Our old answer (below) details why this is the case.*
- *Comparing our method and its results to other published methods is not possible. This is because our method is only able to classify the pollens and objects most commonly found in Quebec organic sediment records. In other words, no public models can classify our target pollens and we cannot classify the target data of other pollen classification models. No labelled dataset suited for our topic currently exist. This is the reason why we had to resort to validating our results using human palynologist predictions (a time intensive process that was necessary in order to achieve any kind of data validation). We thus cannot validate our method on any other public dataset or compare it any further than we already have on recent published methods.*
- *We added a few lines detailed this in the manuscript, and referencing S4 Dataset (Line 140-146; 151-153)*

Comments from reviewer 3

l 21,43 not only environments, but flora & vegetation

- *This has been corrected in the revised manuscript.*

l 54,56 consistency, not consistence

- *This has been corrected in the revised manuscript.*

l 55 All the more - unclear meaning

- *This has been corrected in the revised manuscript. (~~All the more~~ That being said…)*

l 61 ecological processes are inferred, not observed

- *This has been corrected in the revised manuscript. (… allow paleoecologists to ~~quantify~~ infer ecological processes…)*

l 75.76 I think [11-14] is the subject in this sentence

- *This has been corrected in the revised manuscript.*

1 100 full stop needed, not-

- *This has been corrected in the revised manuscript.*

l 155 taxa, not species

- *This has been corrected in the revised manuscript.*

Table 2 Thuja, not Thuya

- *This and other instances have been corrected in the revised manuscript.*

l 169 and throughout pollen, not pollens, Pollen, not Pollens

- *This and other instances have been corrected in the revised manuscript.*

l 179 replace the first P. with Picea, and the third P. with Pinus

- *P. glauca has been changed to Picea glauca. However, we did not change P. banksiana since it had already been fully identified as P. banksiana* Lamb. *earlier, in the first paragraph of the methods.*

l 211, 213 is should be are as data are plural

- *This has been corrected in the revised manuscript.*

l 212 'its' should be 'their'

- *This has been corrected in the revised manuscript.*

l 218 similarity, not similitude

- *This has been corrected in the revised manuscript.*

l 222 add 'the' before least

- *This has been corrected in the revised manuscript.*

l 229 delete of

- *Sentence has been changed: ~~The CNNs' common core architecture comprised of 6 trained layers~~ ->The CNN's common core architecture is comprised of 6 trained layers.*

l 287 precipitation, not precipitations

- *This has been corrected in the revised manuscript.*

l 288 Abies, not A.

- *We did not change this. A. balsamea is referred as Abies balsamea earlier in the text, in section 2.1*

l 289 Alnus, not A.

- *We assume you meant here Acer and not Alnus. In any case, we did not go through with the modification. A. saccharum is referred to as Acer saccharum earlier in the text, in section 2.1.*

l 308 16,319 +-1,975 - is this numbers per cc?

- *Indeed. This has been added in the revised manuscript, on the same line [460].*

l 319 centrifuged, not centrifugated

- *This has been corrected in the revised manuscript.*

l 333 broken-stick is a means of identifying how many zones should (from CONISS or other zonation methods) to be adopted.

- *This is now reworded in order to outline this distinction.*
- *"[…] featuring pollen biozones computed using a statistical cluster analysis zonation method, CONISS. The broken-stick method was used to identify the right number of significant zones to be adopted"*.

l 340 could not, not couldn't

- *This is now corrected in the revised manuscript.*

l 508 Pinus, not P.

- *Again, we did not change this.* Pinus banksiana *is already identified as such in section 2.1.*

l 503 recrudescence - is this a word?

- *This is now corrected in the revised manuscript.*

l 597,598 This should be Birks, HJB & Birks, HH Quaternary Palaeoecology Edward Arnold, London. I am unaware of any connection with the University of California Press

- *Dully noted. This has been fixed in the revised manuscript.*

Below are the review comments from reviewer #2 from the first wave of reviews back in 2023, included as reference:

Comments from reviewer #2:

For example, it is unclear why choosing to "transformed to grayscale and rescaled to 128x128 pixels" (Line 197-8) would standardize the data and improve training performance. This more likely prevented further improvement using RGB colors and larger resolutions. It would be better to just say "we transformed ... in this work".

- *This has been changed in the revised manuscript.*

The choice to limit each CNN's corresponding dataset size to the number of images present in least populated class" (Line 221), throws away a large amount of valuable data. Training on imbalanced data is an established topic in computer vision and there are alternate approaches. A more natural solution would be to adopt weighted loss which assigns a larger weight to rare class.

- *We recognize that neglecting to train the data on imbalanced data was an oversight – we then believed that diving into weighted loss methods would fall outside of the scope of the paper. This is now addressed in the conclusion of the revised manuscript, and will be identified as something to add onto future work.*

Lines 241-246 mentions "transfer learning" but this is actually a discussion of network architecture. The authors should explain how the network was pretrained and on what data, such that the weights can be transferred to pollen data.

- *We included more information on VGG16 and the ImageNet dataset in the revised manuscript. In the quoted paragraph, a few lines already establish how VGG16 can help our own model extract basic features from our data, with references to other papers in automated palynology. We added a line to further explain this, as well as a reference to Alzubaidi et al. (2021).*

The choice to train multiple CNNs was unusual – including the use of a shallow network to train the Alnus CNN because "(deeper models) tend to overfit rapidly" (Line 268). Why did the authors not train a single CNN backbone shared by all classes using all data (allowing learning richer features for all classes)?

- *The inherent taxonomic nature of pollens allowed us to work in a hierarchical classification system (as seen in figure 2). This permits us to have a pollen drop out of the classification and be classified as at the genus level if it falls under a confidence threshold, as seen in Bourel et al. (2020). Similarly to their work, we trained multiple, smaller, CNNs instead of a single one.*
- *Training a single CNN would have us face the issue of an important data imbalance (further addressed in the now revised conclusion) since certain species-level classes had ten times the amount of training data than some others. On the other hand, this allowed us to capitalize on the large amount of training data available when grouping certain species in taxonomic classes. For instance, the top classifier trained on 1,200 images per class.*
- *As proposed in the reviewer comment, something that could have been done is using a CNN trained on all classes and using its base layers in transfer learning. This is now addressed in the revised conclusion.*

Overall, the machine counts did not appear to closely track human/manual counts. The authors make two interesting choices in their comparison. First – human counts are made from images and not from a microscope. This should remove the effect of poor-quality images, as both

human and machine classifications would be affected. But there is still low correlation between the two counts outside of a handful of taxa, e.g. Pinus (Figure 7). Having the human analysis identify detected grains/cropped images doesn't test whether all pollen are identified by the Classifynder, or if there is any systematic bias in what is automatically imaged/detected. Second – given that this is a proof-of-concept paper, why not manually count all 271 samples so that the manual and automatic counts and zonation could be more accurately compared?

- *We indeed chose to validate our model's results using images captured by the Classyfinder microscope. This is addressed in the original manuscript (line 392). In short, this was the only way to isolate our model's own error and bias. What we propose in this paper is not a fully integrated system that requires the Classifynder microscope. Rather, it is a classification tool fit for any fossil pollen sequence whose images have been digitized using any form of light-microscope, automated or not.*
- *Second – while this would have improved the proof-of-concept aspect of our manuscript, counting the full 271 samples would imply counting and identifying a little under 200,000 images. A large part of those images do not belong to any of our trained classes – a single sample can often contain pollen grains and spores from more than 30 species. Since pollen identification is a tedious process and requires a trained specialist, this fell outside of the time and financial limits of the project.*

Based on the images in Figure 3, the discussion of image quality (Lines 526-9) should be significantly expanded. It may be the quality of the images that are the issue and not your models. See the following papers on what is possible with higher quality images:

Punyasena, S. W., Tcheng, D. K., Wesseln, C., & Mueller, P. G. (2012). Classifying black and white spruce pollen using layered machine learning. New Phytologist, 196(3), 937-944.

Kong, S., Punyasena, S., & Fowlkes, C. (2016). Spatially aware dictionary learning and coding for fossil pollen identification. In Proceedings of the IEEE Conference on Computer Vision and Pattern Recognition Workshops (pp. 1-10).

Romero, I. C., Kong, S., Fowlkes, C. C., Jaramillo, C., Urban, M. A., Oboh-Ikuenobe, F., ... & Punyasena, S. W. (2020). Improving the taxonomy of fossil pollen using convolutional neural networks and superresolution microscopy. Proceedings of the National Academy of Sciences, 117(45), 28496-28505.

- *We are thrilled to see how super high-resolution confocal/fluorescence imaging can be integrated into deep learning methods with such good results. We wonder if the above mentioned methods using Zeiss products would be operable for high-volume imaging? Nonetheless, this further shows how imaging may have had a bigger impact on our model than previously thought. We have expanded the discussion of image quality in the revised manuscript.*

L 55: Mander et al 2014 (citation #4) focuses on the classification of grass pollen, which is notoriously difficult to tell apart, even under electron microscopy. It is not the best example of the reproducibility of visual pollen identification and puts traditional palynological data collection in a more negative light than necessary.

- *This is a valid concern. We revised the manuscript in consequence, and referenced other works that touch on the general coherency between analysts.*

L 56: McLeod et al 2010 does not focus on palynology. It also predates the bulk of machine learning research in palynology. There are better references that would characterize the current state of palynological research.

- *We revised the manuscript in consequence; Holt & Bennet (2014) and Mander & Punyasena (2018) give much more accurate insight on the shortcomings of pollen analysis in their respective review papers. Both identify the possible benefits of automated palynology.*

L 65: A number of papers have discussed slide scanning and can be cited here, including:

Holt, K. A., & Bebbington, M. S. (2014). Separating morphologically similar pollen types using basic shape features from digital images: A preliminary study. Applications in Plant Sciences, 2(8), 1400032.

- *In the revised manuscript, cited works related to introducing a deep-learning/slide-scanner workflow, including Holt and Bebbington (2014), Sevillano, Holt & Aznarte (2020) and Pedersen et al (2017).*

L 70, L 90-95: Punyasena et al 2022 (citation #8) also used a slide scanner and image segmentation/pollen detection instead of manual crops. While these were environmental pollen samples (from pollen traps), they have similar issues to Quaternary pollen samples.

- *L70: Added Punyasena et al 2022 to the other citations.*
- *L90-95: Cited Punyasena et al 2022 as an example of combining image detection and an automated slide scanner in the revised manuscript.*

L 173: Were vouchers collected to confirm and record species identifications?

- *We are regretfully unsure of the meaning of this question. The chosen tree species are handily identifiable in the wild and are not known to present a challenge in the matter.*

L 190: Provide more detail on how “pollen was automatically identified through the use of

morphological criteria input by the user”. Is this using the Classyfinder software? If so, explain how this is automated and what specific criteria are used.

- *This was using the Classyfinder software. We added the specific criteria in the SI.*

L 192: Are you using a Z-stack (multiple images) of pollen specimen or a single fused image? This is not clear.

- *This was using a single fused image. Line has been reworked to reflect this in the revised manuscript.*

L 208: The GitHub repository mentions that the Python code should work with PyTorch. Might be worth mentioning that here too.

- *Added to the revised manuscript.*

L 241: What is meant by the “bottom” of the model? The initial layers?

- *The initial layers. Line has been revised in the new manuscript.*

L 299: Using different processing methods for reference pollen, fresh pollen, and fossil pollen introduces visual artifacts that makes the classification problem much harder than it should be. Ideally, as many variables as possible should be controlled. The authors should consider comparing like with like in their training and testing and explicitly test whether processing choices affected the results.

- *There is regretfully no way around processing the fresh and fossil pollens differently. This is addressed in section 2.4, as well as what laboratory methods were used to minimize discrepancy. The goal of this project was to create a model capable of classifying fossil pollens extracted from a sediment core. The fossil pollens unavoidably undergo a different processing method than fresh pollens do. This is mostly in order to flush out siliceous material (dense-media separation, sodium polytungstate), de-clump the pollens and burn the remaining organic particles (filtering, KOH and ultrasonic bath). Most of the remaining visual artifacts were filtered out using the Main CNN, which has a very accurate NPP/mineral class. Fresh pollens do not need to undergo such a process – instead, some of them require to have their inside cleaned out of cellular content using either enzymes or acetolysis.*

- *We do not believe that training on 14 classes (and 18 species) would have been possible using fossil pollens – this would imply a point and click method of gathering data going through a fossil pollen slide. This would not have allowed us to gather as much data. Furthermore, a training set composed of fossil pollens found on a slide would add bias:*

only the pollens that are in good condition with apparent features would have been chosen, in order to avoid wrongly labeling a pollen.

- *Using fresh pollens allowed us to train the model on considerably more images than what we could have gotten by individually identifying and labelling fossil pollens. This also allowed us to collect data of heavily damaged pollen (of such that is commonly found in organic (lacustrine) sediments, for instance the often found saccus of an unknown* Pinus *or* Picea *grain floating about). Such pollens would have otherwise proved to be impossible to visually identify and add to the training set (Does this saccus belong to a* P. banksiana*,* P. strobus or P. resinosa?).
- *This rationale has been added to the revised manuscript (Line 118) The lab protocols can be found in sections 2.2 and 2.4 and we invite other researchers to experiment and improve it.*

L 326: As per the comment above, any visual documentation of the differences in this alternate processing method?

- *We believe this falls outside the scope out the paper and would only burden and already elaborate manuscript. We do however plan on submitting this lab protocol, along with visual documentation, in the future – we believe its output is impressive (30 organic sediment samples in a day is higher than our usual 16-20) and would benefit from being be tested in other labs.*

L 553: The word "recrudescence" has negative connotations. Use "re-emergence" or "reappearance" instead.

- *This has been changed in the revised manuscript.*

Recurring: Recommend using a spelling/grammar check to catch errors. Species and genera names should be italicized.

- *We understand that this italicizing (or lack) may be an issue. We made sure to only italicize species and genera names – the names of classes are not. This helps differentiate when we write about the species or their respective model classes. Model classes are often comprised of multiple species but are labelled as being one, as is sometimes done in palynology: for instance, the Picea class is made up of Picea glauca and Picea rubens.*
- *We added a disclaimer in the description of Table 2.*

Figure 3: Include example images of all 13 classes.

- *The figure has been updated in the revised manuscript, along with the figure legend.*

Figures 8 and 9: It would be helpful to see zonations overlaid or side by side.

- *Figure 9's zonations are already overlaid on figure 8 (dotted-lines). This is stated in the figure description.*
