# Supplementary material for: Pollen identification through convolutional neural networks: First application on a full fossil pollen sequence
Source: PLoS One. 2024 Apr 30;19(4):e0302424. doi: 10.1371/journal.pone.0302424 (PMC11060525; doi:10.1371/journal.pone.0302424)
Supplement: Attachment — Submitted filename: PONE-D-23-19374R2_authors.docx [file pone.0302424.s005.docx]

PONE-D-23-19374R2

This is a thorough and convincing ‘proof of concept’ (as the authors conclude) study. It contains several interesting ideas and methods and adds to the rapidly growing literature on attempts to achieve automated pollen counting. However, the very recent publication by von Allmen et al. (2024, Quaternary Science Reviews 327; 108521) is a ‘proof of principle’ (as the authors describe their study). It certainly leads the field in showing what convolutional neural networks in automated palynology can or could achieve.

Concerning the manuscript by Durand et al. submitted to PLoS ONE, I recommend it be accepted after some editorial corrections, etc. as given below.

Lines 20, 37, 58 and throughout: Perhaps better to use ‘identification’ than ‘classification’?

Line 66: Change ‘accurate’ to ‘accuracy’

Line 70: See the very recent paper by von Allmen et al. in QSR 327: 108521.

Line 101: Also the presence of organic debris

Line 129: Replace *A.* with *Acer*; replace *P.* with *Pinus* (when there is potential confusion, or starting a sentence with the species, always put the genus name in full. Here, *A*. is used for both *Acer* and *Abies*, *P.* is both *Picea* and *Pinus*, so the abbreviation can only be used in close proximity to the full name).

Line 130: Replace *B.* with *Betula*; replace *A.* with *Abies*

Line 140: Not the first now, see von Allmen et al. (2024, QSR)

Line183/4: I do not understand how calling a four- or five-pored pollen type triporate streamlines the classification process.

Line 215: Change ‘is’ to ‘are’ after data

Line 220/2: It seems to me to be a real weakness that the test and validation datasets are from the same dataset as the training data, namely CNN.

Line 234: Change ‘fail’ to ‘fails’

Line 271: Are there 14 million images of pollen?

Line 317: Replace *A.* with *Abies*

Line 318: Replace *B.* with *Betula*

Line 319: Replace *A.* with *Acer*

Line 339: And pollen concentration (n cm^–3^)

Line 375: Change ‘certitude’ to ‘certainty’

Line 390: Pre- and post-calibration?

Line 404: Add ‘to’ after ‘next’

Line 425: Change ‘limit’ to ‘limitation’

Line 454: Under- or over-predictions?

Line 482: Replace *J.* with *Juniperus*

Line 482/3: How can one say that Unknown/Other pollen is extra-local?

Line 584: Change ‘reconstruction’ to ‘sequence’

Line 630: Should this be CA Lucas?

Line 680/1: Reference 16 needs amending – ‘In Sydney, Australia’

Line 692: Should it say Melbourne, Florida rather than Melboure, Floride?

Fig. 2: Difficult to read the text in the green parts.
