# Supplementary material for: Pollen identification through convolutional neural networks: First application on a full fossil pollen sequence
Source: PLoS One. 2024 Apr 30;19(4):e0302424. doi: 10.1371/journal.pone.0302424 (PMC11060525; doi:10.1371/journal.pone.0302424)
Supplement: Attachment — Submitted filename: PONE-D-23-19374R2_eds.docx [file pone.0302424.s006.docx]

PONE-D-23-19374R2

To Editor,

I have no competing interests.

I have read the revised version of this manuscript. It is considerably better than the earlier version I reviewed. There are minor editorial corrections needed, as I have listed in Comments to the authors.

My recommendation is Accept after minor revision.
